# JMJD2A promotes the Warburg effect and nasopharyngeal carcinoma progression by transactivating LDHA expression

**DOI:** 10.1186/s12885-017-3473-4

**Published:** 2017-07-11

**Authors:** Yi Su, Qiu-hong Yu, Xiang-yun Wang, Li-ping Yu, Zong-feng Wang, Ying-chun Cao, Jian-dong Li

**Affiliations:** 1 Department of E.N.T., Dongying People’s Hospital, Shandong, 257091 China; 2 Department of E.N.T., Kenli People’s Hospital, Shandong, China

**Keywords:** Nasopharyngeal carcinoma, Jumonji C domain 2A, LDHA, Glycolysis

## Abstract

**Background:**

Jumonji C domain 2A (JMJD2A), as a histone demethylases, plays a vital role in tumorigenesis and progression. But, its functions and underlying mechanisms of JMJD2A in nasopharyngeal carcinoma (NPC) metabolism are remained to be clarified. In this study, we investigated glycolysis regulation by JMJD2A in NPC and the possible mechanism.

**Methods:**

JMJD2A expression was detected by Western blotting and Reverse transcription quantitative real-time PCR analysis. Then, we knocked down and ectopically expressed JMJD2A to detect changes in glycolytic enzymes. We also evaluated the impacts of JMJD2A-lactate dehydrogenase A (LDHA) signaling on NPC cell proliferation, migration and invasion. ChIP assays were used to test whether JMJD2A bound to the LDHA promoter. Finally, IHC was used to verify JMJD2A and LDHA expression in NPC tissue samples and analyze their correlation between expression and clinical features.

**Results:**

JMJD2A was expressed at high levels in NPC tumor tissues and cell lines. Both JMJD2A and LDHA expression were positively correlated with the tumor stage, metastasis and clinical stage. Additionally, the level of JMJD2A was positively correlated with LDHA expression in NPC patients, and higher JMJD2A and LDHA expression predicted a worse prognosis. JMJD2A alteration did not influence most of glycolytic enzymes expression, with the exception of PFK-L, PGAM-1, LDHB and LDHA, and LDHA exhibited the greatest decrease in expression. JMJD2A silencing decreased LDHA expression and the intracellular ATP level and increased LDH activity, lactate production and glucose utilization, while JMJD2A overexpression produced the opposite results. Furthermore, JMJD2A could combine to LDHA promoter region and regulate LDHA expression at the level of transcription. Activated JMJD2A-LDHA signaling pathway promoted NPC cell proliferation, migration and invasion.

**Conclusions:**

JMJD2A regulated aerobic glycolysis by regulating LDHA expression. Therefore, the novel JMJD2A-LDHA signaling pathway could contribute to the Warburg effects in NPC progression.

**Electronic supplementary material:**

The online version of this article (doi:10.1186/s12885-017-3473-4) contains supplementary material, which is available to authorized users.

## Background

Nasopharyngeal carcinoma (NPC) is arising from the nasopharynx epithelium. Although NPC has a low morbidity worldwide, its geographical distribution pattern is very unique. Though the incidence of NPC in all cancers are only 0.6% diagnosed in one year, yet 71% of new patients appeared in the east and southeast of Asia [1, 2]. Although a decrease in the incidence and a substantial reduction in mortality have been observed due to the early diagnosis of NPC and advanced radiotherapy and chemotherapy, unsatisfactory outcomes remain for patients with locally advanced and metastatic NPC. Therefore, studies identifying novel and specific biomarkers for NPC are critically important and urgently needed, with the hope of improving NPC patient’s prognosis. In addition to regular genetic regulation, epigenetic modification plays a vital role in NPC, particularly in gene methylation [3] and histone methylation [4]. However, histone demethylation, have been remained immensely uncovered in NPC mechanisms.

The majority of histone demethylases belong to the Jumonji C domain-containing (JMJD) proteins family [5]. JMJD2A belongs to this family and is capable of demethylating H3K9 and H3K36 [6]. It is overexpressed in many types of cancers, such as prostate [7], breast [8–10] and lung [11] cancers, promoting tumor progression. Dependency on aerobic glycolysis, is a highlighting hallmark of cancers, as known as the Warburg effects [12]. Abnormal glycolysis was recently observed in NPC cells and was associated with a poor prognosis for NPC patients [13, 14]. Additionally, metabolic reprogramming orchestrates cancer stem cell properties, promoting NPC development and progression [15].

Here, we show how JMJD2A exerted its cellular functions through the Warburg effect by interacting with a key element of glycolysis, lactate dehydrogenase A (LDHA). To our knowledge, we are also the first to report that high levels of JMJD2A expression may be a possible cause of NPC tumorigenesis and might be a prognostic marker for patients with NPC. Therefore, JMJD2A should be highlighted as a valid anticancer drug target.

## Methods

### Human tissue specimens

Fifty cases of NPC samples and 20 normal controls were collected from the E.N.T. Department, Dongying People’s Hospital, Shandong Province, from July 2002 to December 2012. All the patients were diagnosed and verified of NPC by histology, without receiving radiotherapy or chemotherapy. We collected the clinicopathological features of patients with NPC, and the follow-up concluded in January 2017. The research was approved and supervised by the Research Ethics Committee of Dongying People’s Hospital, Shandong Province, China, and the written consent had been obtained from all the NPC patients.

We used xylene to deparaffinize the tissue samples and then rehydrated then in a series of graded alcohol solutions. Endogenous peroxidases were blocked with 3% H_2_O_2_, and antigens were retrieved by heating the samples in citrate buffer. We then incubated the tissue samples with a rabbit antibody against JMJD2A (dilution 1:100; CST, Cambridge, UK) or LDHA (dilution 1:400; CST, Cambridge, UK) overnight at 4 °C followed by horseradish peroxidase (Gene Tech GTVision III Detection Kit, Shanghai, China) for 40 min at room-temperature. Then washing the sections with PBS buffer for 3 times, and testing the signal by a DAB solution.

### Scoring of the immunohistochemistry (IHC)

A double-blind method was used to analyze the IHC results: two pathologists without access to the patients’ clinical and pathological characteristics independently evaluated the results. Five different areas of visual fields selected from each specimen were randomly chosen for the immunohistochemical evaluation. JMJD2A and LDHA expression were scored by the percentage of positive cells as well as the staining intensity as previously described [16, 17]. The IHC scorings were as follows: 0, no positive cells; 1, ≤10% positive cells; 2, 10–50% positive cells; and 3, >50% positive cells; 0, no staining; 1, faint staining; 2, moderate staining; and 3, dark staining. Comprehensive scores = staining percentage × intensity. JMJD2A and LDHA expression were classified as follows: ≤2 low expression or >2 high expressions.

### Cell lines and reagents

The nasopharyngeal epithelial cell lines NP69 (ATCC-5859) and NPC cell lines CNE2 (ATCC-1434), CNE1(ATCC-0364), HONE1(ATCC-0369), HNE1(ATCC-0366), 5-8F (ATCC-2496) and 6-10B (ATCC-6605) were obtained from Jennio-bio (Guangzhou, China). NP69 cells were cultivated in keratinocyte/serum-free medium (Invitrogen, Carlsbad, CA, USA) supplemented with EGF (epidermal growth factor, Invitrogen). All NPC cell lines were cultured in RPMI 1640 medium ((Gibco, Rockville, MD, USA)) supplemented with 10% FBS (HyClone, Logan, UT, USA). All the cell lines were incubated at 37 °C with the humidity of 5% CO_2_ atmosphere_._ Oxamate (Oxa) sodium was bought from Sigma-Aldrich Corp. (St. Louis, MO, USA). Oxa was dissolved and diluted in the sterile water, and the final concentration was achieved by diluting Oxa in culture medium, which was phenol-red-free RPMI with 1% FBS.

### Plasmids construction and small interfering RNAs

The control vector pcDNA3.1 and plasmids pcDNA3.1-JMJD2A (pJMJD2A) was described previously [18]. A small interfering RNA (siRNA) targeting JMJD2A (siJMJD2A) (GenePharma, Shanghai, China) was used to decrease its expression. The sequences were as follows: Sense: 5′ GUAUGAUCUUCCAGACUUA 3′ and Antisense: 5′ UAAGUCUGGAAGAUCAUAC 3′.

### Transient transfection

We used Lipofectamine 2000 and Lipofectamine RNAiMAX (Invitrogen, Grand Island, NY, USA) to transfect plasmids and siRNAs into NPC cells lines, respectively. For transient transfections, NPC cells were transfected with the indicated plasmids or siRNAs for 24 or 48 h prior to the functional assays or WB assays, respectively. NPC cells transfected with empty vectors were defined as control groups, and untreated cells were defined as mock groups.

### RNA extraction and Reverse transcription quantitative real-time PCR analysis

We used TRIzol reagent (Invitrogen; Thermo Fisher Scientific, Inc.) to extract total RNA from tissue samples and cells lines, according to the manufacturers’ protocol. The extracted RNA was tested and quantified by ultraviolet spectrophotometry. Then the quantified RNA was reversely transcribed into cDNAs by ExScript RT-PCR kit (TaKaRa Bio, Inc., Otsu, Japan). Then, quantitative real-time PCR analysis was used to detect the targeted genes expression. GAPDH was used as an internal control. The primer sequences are listed in Table 1. Comparative threshold cycle (Ct) (2^-ΔΔCt^) method was used to calculate the gene relative mRNA expression.

**Table 1:** The primer sequences of glycolytic enzyme

Name	Abbreviation	Primers
Jumonji domain containing 2A	JMJD2A	Sense: 5′-ATCCCAGTGCTAGGATAATGACC-3′
		Anti-sense: 5′-ACTCTTTTGGAGGAACCCTTG-3′
Glucose transporter-1	GLUT-1	Sense: 5′-CTTTGTGGCCTTCTTTGAAGT-3′
		Anti-sense: 5′-CCACACAGTTGCTCCACAT-3′
Glucose transporter-4	GLUT-4	Sense: 5′-CTTCATCATTGGCATGGGTTT-3′
		Anti-sense: 5′-CGGGTTTCAGGCACTTTTAGG-3′
Hexokinase-II	HK-II	Sense: 5′-GATTTCACCAAGCGTGGACT-3’
		Anti-sense: 5′-CCACACCCACTGTCACTTTG-3′
Glucose-6-phosphate isomerase	G6PI	Sense: 5′-AGGCTGCTGCCACATAAGGT-3′
		Anti-sense: 5′-AGCGTCGTGAGAGGTCACTTG-3′
Muscle-type phosphofructokinase	PFK-M	Sense: 5′-ATTCGGGCTGTGTTCTGG-3′
		Anti-sense: 5′-TGGCTAGGATTTTGAGGATGG-3′
Liver-type phosphofructokinase	PFK-L	Sense: 5′-GGACAGGAAAGAGGAAGTGAC-3′
		Anti-sense: 5′-CGTAGATGAGGAAGACTTTGGC-3′
Platelet isoform of phosphofructokinase	PFK-P	Sense: 5′-CATCGACAATGATTTCTGCGG-3′
		Anti-sense: 5′-CCATCACCTCCAGAACGAAG-3′
Aldolase B	AldoB	Sense: 5′-ATGCCACTCTCAACCTCAATGCTATC-3′
		Anti-sense: 5′-TTATTTTCTTGGGTGGGTATTCTGG-3′
Phosphoglycerate kinase 1	PGK-1	Sense: 5′ -CGGTAGTCCTTATGAGCC-3′
		Anti-sense: 5′-CATGAAAGCGGAGGTTCT-3′
Phosphoglycerate mutase 1	PGAM-1	Sense: 5′-CCTGGAGAACCGCTTC-3′
		Anti-sense: 5′-CATGGGCTGCAATCAGTACAC-3′
Enolase	Enolase	Sense: 5′-CTGATGCTGGAGTTGGATGG-3′
		Anti-sense: 5′-CCATTGATCACGTTGAAGGC-3′
M1 isoform of pyruvate kinase	PKM1	Sense: 5′-CTATCCTCTGGAGGCTGTGC-3′
		Anti-sense: 5′-CCATGAGGTCTGTGGAGTGA-3′
M2 isoform of pyruvate kinase	PKM2	Sense: 5′-GGGTTCGGAGGTTTGATG-3′
		Anti-sense: 5′-ACGGCGGTGGCTTCTGT-3’
Lactate dehydrogenase B	LDHB	Sense: 5′-CCTAGAGCTCACTAGTCACAG-3′
		Anti-sense: 5′-CTCCTGTGCAAAATGGCAAC-3′
Lactate dehydrogenase A	LDHA	Sense: 5′-CAGCTTGGAGTTTGCAGTTAC-3′
		Anti-sense: 5′-TGATGGATCTCCAACATGG-3′
Glyceraldehyde-3-phosphate dehydrogenase	GAPDH	Sense: 5′-TGACGCTGGGGCTGGCATTG-3′
		Anti-sense: 5′-GCTCTTGCTGGGGCTGGTGG-3′

### Western blotting analysis

Standard Western blotting was conducted using proteins from whole cells lysed in RIPA buffer, and using primary antibodies against JMJD2A, LDHA and GAPDH, and indicated secondary antibody.

### LDH activity, lactate production, glucose utilization assay and the intracellular ATP level

NPC cells were transfected transiently with plasmids and siRNAs, with/without treatment of oxamate (20 mmol/L). 1 × 10^6^ cells were used to test LDH activity. Lactate production was detected by Lactate Dehydrogenase Activity Assay Kit and Lactate Assay Kit (Sigma, St. Louis, MO, USA). For glucose utilization assay, NPC cells were transiently transfected. After 24 h, phenol-red free RPMI supplemented with 1% FBS or with 1% FBS and 20 mmol/L oxamate replaced the previous media, and cultured for 72 h. A colorimetric glucose assay kit (BioVision, Milpitas, CA, USA) was supplied to measure the glucose concentrations [19]. Intracellular ATP levels were detected using a firefly luciferase-based ATP assay kit (Beyotime Institute of Biotechnology, Haimen, China). The protein concentration was also tested using a BCA protein assay kit (Beyotime Institute of Biotechnology, Haimen, China). The relative ATP level is expressed as the ATP concentration/protein concentration.

### Chromatin Immunoprecipitation (ChIP) assay

ChIP assays were performed using cell lines DNA by ChIP kit purchased from CST. Briefly, about 5 × 10^6^ cells were treated with 1% formaldehyde aimed for cross-linking procedure, and the reaction was then stopped by the adding 0.125 M glycine. The NPC cells were scraped and collected after centrifugation at the speed of 800 g for 5 min at 4 °C. Next, the cross-linked segments were resuspended using SDS lysis buffer which contained protease inhibitor cocktail II, and the soluble chromatin was pieced to fragment the DNA using nuclear lysis buffer. The fragmented chromatin were aliquoted, each as genomic input DNA or immunoprecipitated with JMJD2A or IgG antibodies. The mixed solutios were incubated at 4 °C with rotation overnight. The complexes were collected with a magnetic separator, followed by washing and eluting with ChIP elution buffer. The spin columns were used to purify DNA. The ChIP products and genomic input DNA were analyzed by Reverse transcription quantitative real-time PCR analysis. The three pairs of LDHA primers used for ChIP assays were the following:

- sense, 5′-caagccactgacagttcttg-3′
- antisense, 5′-ACCTAAGTCGAGTGACCTCC-3′
- sense, 5′-GTGCTATTTTGGAGCTGAGGTT-3′
- antisense, 5′-AGCCCTTGAGTATGCCAAAAT-3′
- sense, 5′-TATCTCAAAGCTGCACTGGGC-3′,
- antisense, 5′-TGCTGATTCCATTGCCTAGC-3′

### MTT assay

A MTT assay was performed to evaluate cell proliferation ability. About 5000 cells were seeded into each wells and transfected with plasmid and followed by the treatment with or without oxamate sodium (20 mmol/L) with 24, 48 and 72 h, relatively. Next, 5 mg/L of the MTT solution was added to each wells, and incubated at 37 °C for 4 h. Discarding the supernatant and adding 150 μL DMSO for disolving. At last, the absorbances were measured by a microplate reader (Bio-Tek Instruments, Inc., Winooski, VT, USA) at the wave length of 570 nm.

### NPC cell migration and invasion assays

NPC cells were transfected with control, siJMJD2A or pJMJD2A. 1 × 10^5^ cells in 600 μL of serum-free medium were placed in the upper chamber with or without a Matrigel-coated membrane (Millipore, Billerica, MA, USA). RPMI-1640 supplemented with 10% FBS or with 10% FBS and 20 mmol/L oxamate place in the lower chamber was used as a chemoattractant.

### Promoter reporter construction and dual luciferase assay

A fragment containing the sequences from −1330 to +150 bp of the LDHA gene relative to the transcription initiation site was subcloned into the pGL3-basic vector [20] (the vector was constructed and verified by Obio Bioengineering Co., Ltd.). The NPC cells were transfected by the constructed LDHA promoter reporter, siJMJD2A, or overexpression plasmid. Co-transfecting a β-actin/Renilla luciferase reporter, which includes a full-length Renilla luciferase gene, was used as normalizing the LDHA promoter activity. A dual luciferase assay kit (Promega, Madison, WI, USA) was employed to detect the luciferase activity in 24 h after transfection.

### Statistical analysis

All data are measured and presented as means ± standard deviations. Two groups were compared using Student’s t-test, whereas three or more groups were compared using one-way analysis of variance with SPSS 13.0 (SPSS, Inc., Chicago, IL, USA). The analysis of the correlations between the clinicopathological characteristics and JMJD2A and LDHA expression was using χ^2^ test. A Univariate and Cox regression analysis was also performed. The Kaplan-Meier method was used to assess overall survival. A value of *P* < 0.05 was considered statistically significant.

## Results

### JMJD2A is expressed at high levels in NPC

**Fig. 1:**
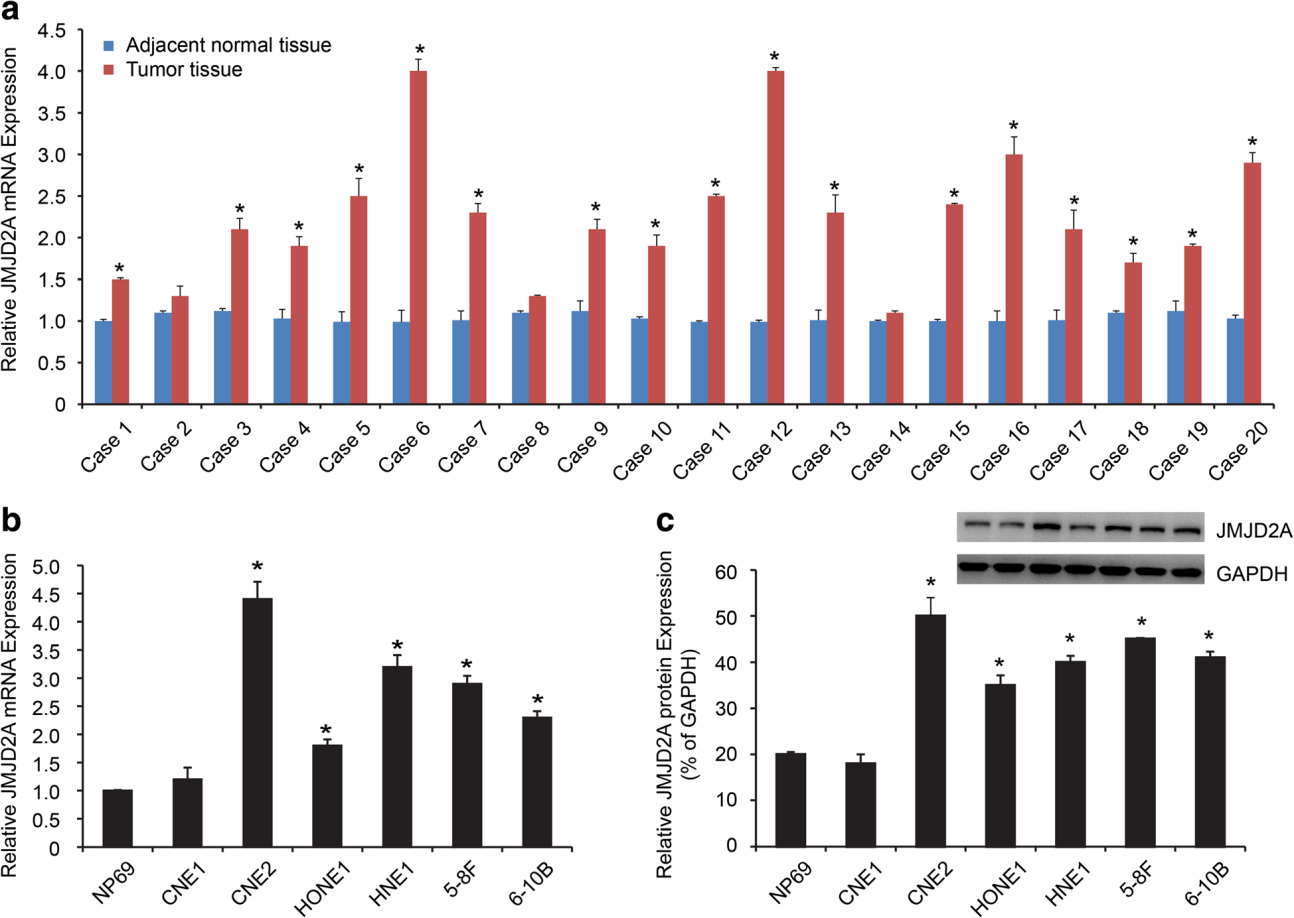
JMJD2A expression in NPC tissue samples and cell lines. **a** qRT-PCR was performed to analyze the expression of the JMJD2A mRNA in NPC tumor tissues and adjacent normal tissues. **b** qRT-PCR was employed to analyze the expression of the JMJD2A mRNA in NPC cell lines and the normal epithelial cell line NP69. **c** Western blotting analysis was used to detect JMJD2A protein in NPC cell lines and the normal epithelial cell line NP69. *P < 0.05

Twenty pairs of NPC tissues and corresponding normal tissues samples were used to detect the JMJD2A mRNA expression. Seventeen of twenty NPC tissues showed significantly higher JMJD2A expression (Fig. 1a; *P* < 0.05). We also used cell lines to confirm this result. Compared with NP69, the other NPC cell lines showed higher expression of both the JMJD2A mRNA and protein (Fig. 1b and c; *P* < 0.05).

### JMJD2A upregulates LDHA expression in NPC

**Fig. 2:**
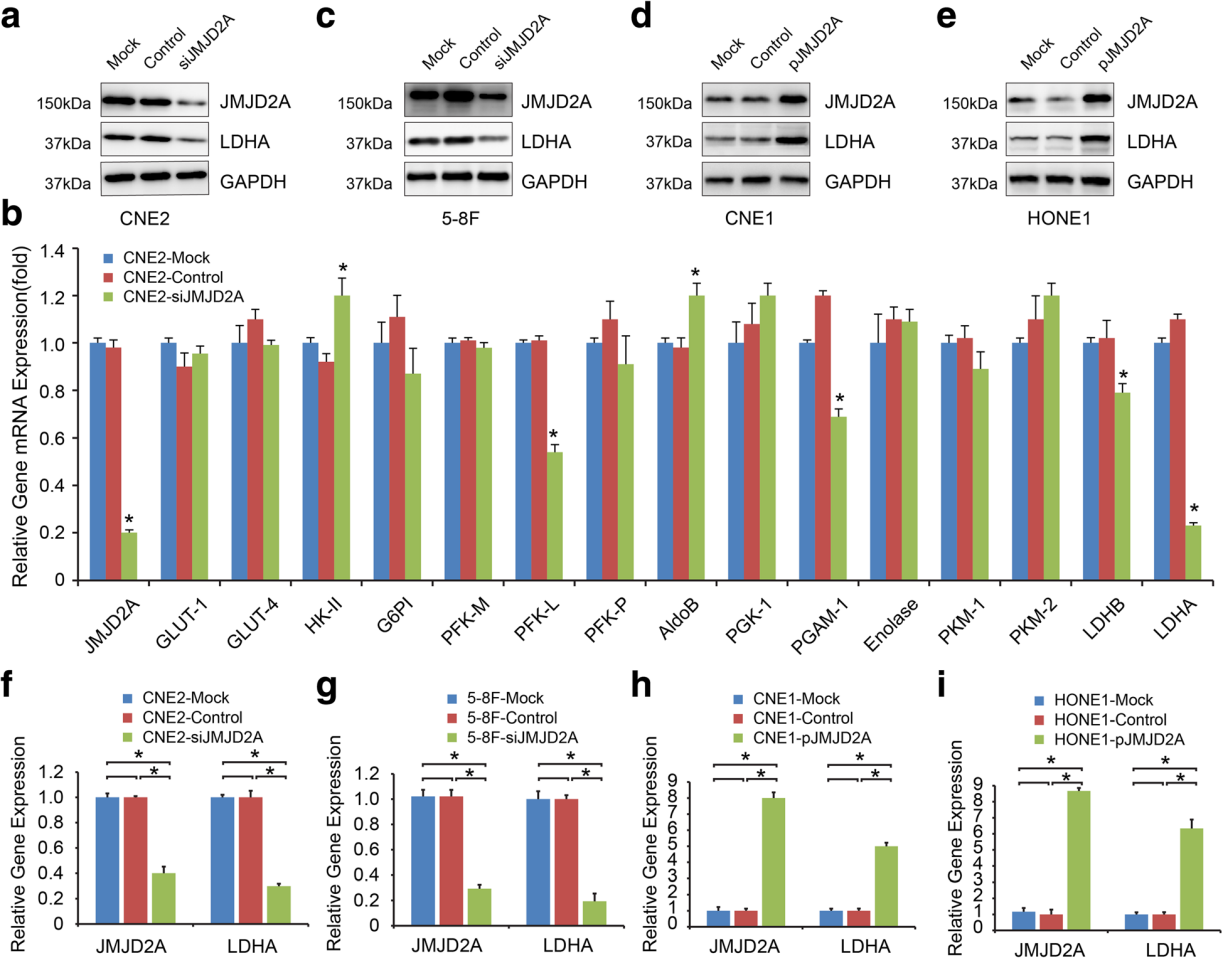
JMJD2A upregulates LDHA expression in NPC. **a** Knockdown of JMJD2A decreased LDHA expression in CNE2 cell lines. **b** The levels of glycolytic enzyme mRNAs in JMJD2A-silenced cells were assessed using qRT-PCR. **c** Knockdown of JMJD2A decreased LDHA expression in 5-8F cell lines. **d-e.** Overexpression of JMJD2A increased LDHA expression in CNE1 (D) and HONE1 (E) cell lines. **f-i** JMJD2A and LDHA mRNA levels in CNE2 (F), 5-8F (G), CNE1 (H) and HONE1 (I) cells with altered levels of JMJD2A were detected by qRT-PCR. *P < 0.05

Aerobic glycolysis is the major feature for cancer metabolism; thus, we paid attention to the regulatory effects of JMJD2A on glycolysis in NPC cells. We assessed the effects of JMJD2A on the glycolytic enzymes expression. We first used RNAi to knockdown JMJD2A expression and confirmed the knockdown efficacy (Fig. 2a; *P* < 0.05). Next, we used CNE2-siJMJD2A cells to detect the levels of glycolytic enzyme mRNAs by qRT-PCR. JMJD2A silencing did not alter the most of the enzymes, with the exception of the downregulation the expression of PFK-L, PGAM-1, LDHB and LDHA (Fig. 2b *; P* < 0.05). Among the enzymes listed above, LDHA exhibited the greatest decrease in expression. We then confirm the effects of JMJD2A expression on the LDHA protein. We used CNE2 and 5-8F cells, which express higher levels of JMJD2A, to verify the siJMJD2A results and found that JMJD2A silencing downregulated LDHA expression (Fig. 2a and c; *P* < 0.05). Meanwhile, LDHA expression was upregulated in the low-JMJD2A expressing cell lines CNE1 and HONE1 that had been transfected with the JMJD2A expression plasmid (Fig. 2d and e). These results were also confirmed at the mRNA level by qRT-PCR (Fig. 2f, g, h, and i; *P* < 0.05).

### JMJD2A activates LDHA expression at transcriptional level by in NPC cells

**Fig. 3:**
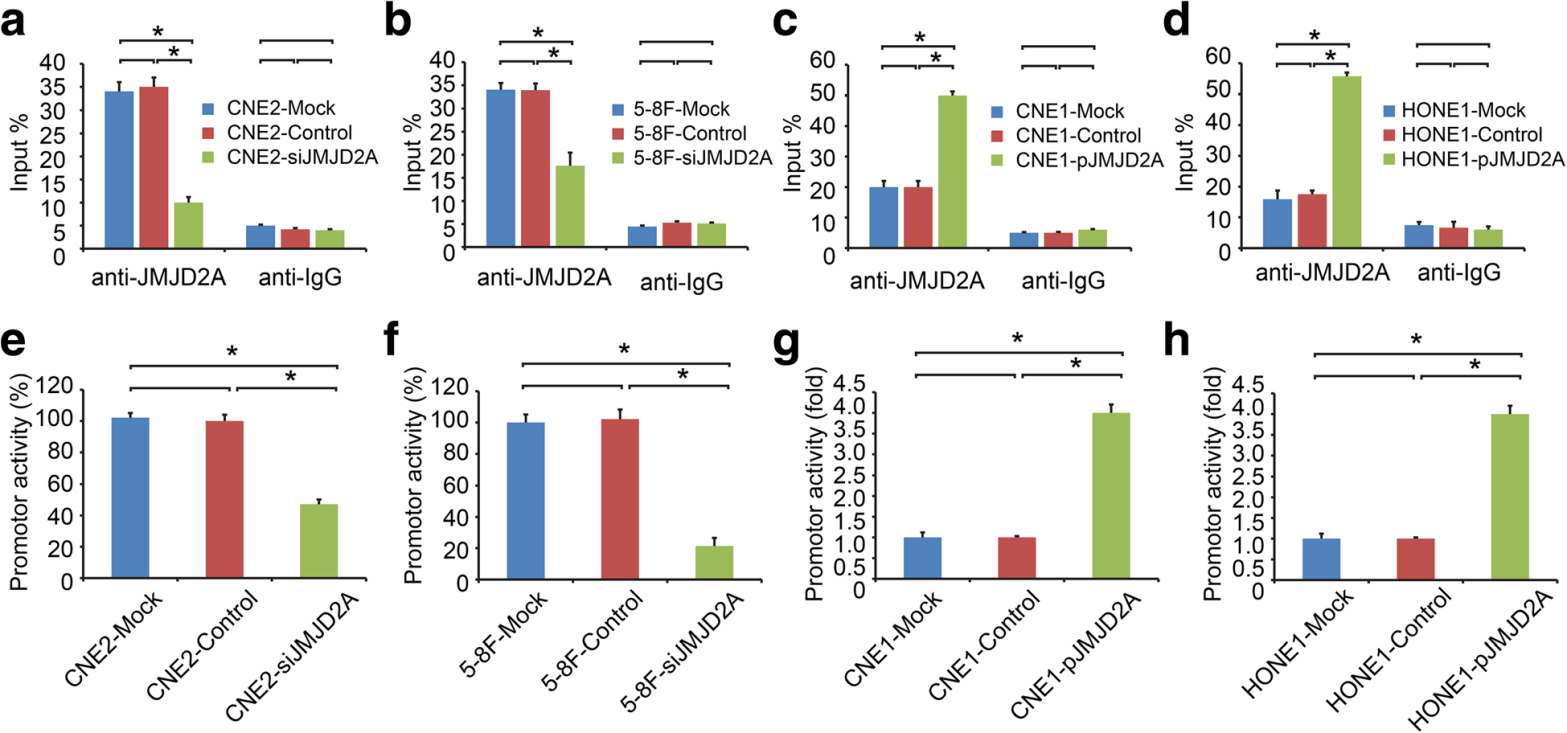
Transcriptional activation of LDHA expression by JMJD2A in NPC cells. **a-b** Chip assays revealed that JMJD2A knockdown decreased the binding of JMJD2A to the LDHA promoter in CNE2 (A) and 5-8F (B) cell lines. **c-d** JMJD2A overexpression increased JMJD2A binding to the LDHA promoter in CNE1 (C) and HONE1 (D) cells. **e-h** Luciferase reporter assay were performed to detect the effect of JMJD2A on LDHA transcription. Silencing of JMJD2A decreased LDHA promoter activity in CNE2 (E) and 5-8F (F) cell lines, whereas JMJD2A overexpression elevated the LDHA promoter activity in CNE1 (G) and HONE1 (H) cell lines. *P < 0.05

Our data revealed a direct correlation between JMJD2A and LDHA expression. ChIP assay were conducted to further explore the mechanisms by which JMJD2A regulated LDHA expression. We designed three pairs of primers targeting the LDHA promoter region **(**Additional file 1: Figure S1A). JMJD2A bound to the LDHA promoter, and among the primers, primer b showed the largest difference (Additional file 1: Figure S1B; *P* < 0.05). Thus, the subsequent investigations were only performed using primer b. We used both the overexpression and RNAi systems to confirm the results and found that JMJD2A knockdown decreased the binding of JMJD2A to the LDHA promoter in the CNE2 and 5-8F cell lines, leading to the downregulation of LDHA expression (Fig. 3a and b; *P* < 0.05). Meanwhile, JMJD2A overexpression increased JMJD2A binding to the LDHA promoter in the CNE1 and HONE1 cell lines, leading to the upregulation of LDHA expression (Fig. 3c and d; *P* < 0.05). Then, a luciferase reporter assay was to detect the effects of JMJD2A on LDHA transcription. Silencing of JMJD2A decreased LDHA promoter activity (Fig. 3e and f; *P* < 0.05), whereas JMJD2A overexpression elevated the LDHA promoter activity (Fig. 3g and h; *P* < 0.05). Based on these data, JMJD2A bound to the LDHA promoter and activated LDHA expression transcriptionally. Further study will be emphasized on looking for possible transcriptional factors that bind to JMJD2A and directly interact with the LDHA promoter.

### JMJD2A promotes the Warburg effect in NPC cells

**Fig. 4:**
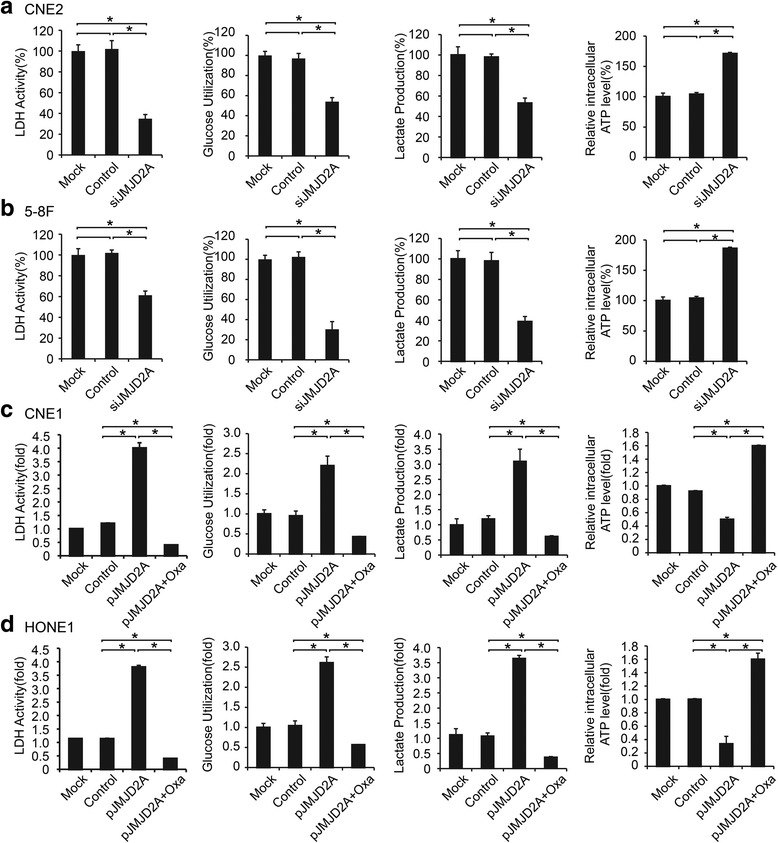
JMJD2A promoted the Warburg effect in NPC cells. **a-b** LDHA activity, glucose utilization, lactate production, and increase in intracellular ATP levels were assessed in CNE2 (A) and 5-8F (B) cell lines transfected with siJMJD2A. **c-d** LDHA activity, glucose utilization, lactate production, and increase in intracellular ATP levels were assessed in CNE1 (C) and HONE1 (D) cell lines transfected with pJMJD2A and treated with or without 20 mmol/L oxamate sodium. *P < 0.05

Because we have observed that JMJD2A is associated with LDHA expression, we further explored the impact of JMJD2A on the Warburg effect, including LDH activity, glucose utilization, lactate production, and the intracellular ATP level. After knocking down JMJD2A, we observed significant decreases in LDH activity, glucose utilization, and lactate production, as well as an increase in the intracellular ATP level (Fig. 4a and b; *P* < 0.05). In comparison, JMJD2A upregulation markedly increased LDH activity, lactate production, glucose utilization, and also decreased the intracellular ATP level in cells (Fig. 4c and d; *P* < 0.05). LDHA activity inhibition by oxamate sodium attenuated the JMJD2A-induced increase in glucose utilization, lactate production, and LDH activity (Fig. 4c and d; *P* < 0.05). Thus, JMJD2A may regulate lactate production and glucose utilization by regulating LDHA activity.

### JMJD2A-LDHA signaling promotes NPC cell proliferation, migration and invasion

**Fig. 5:**
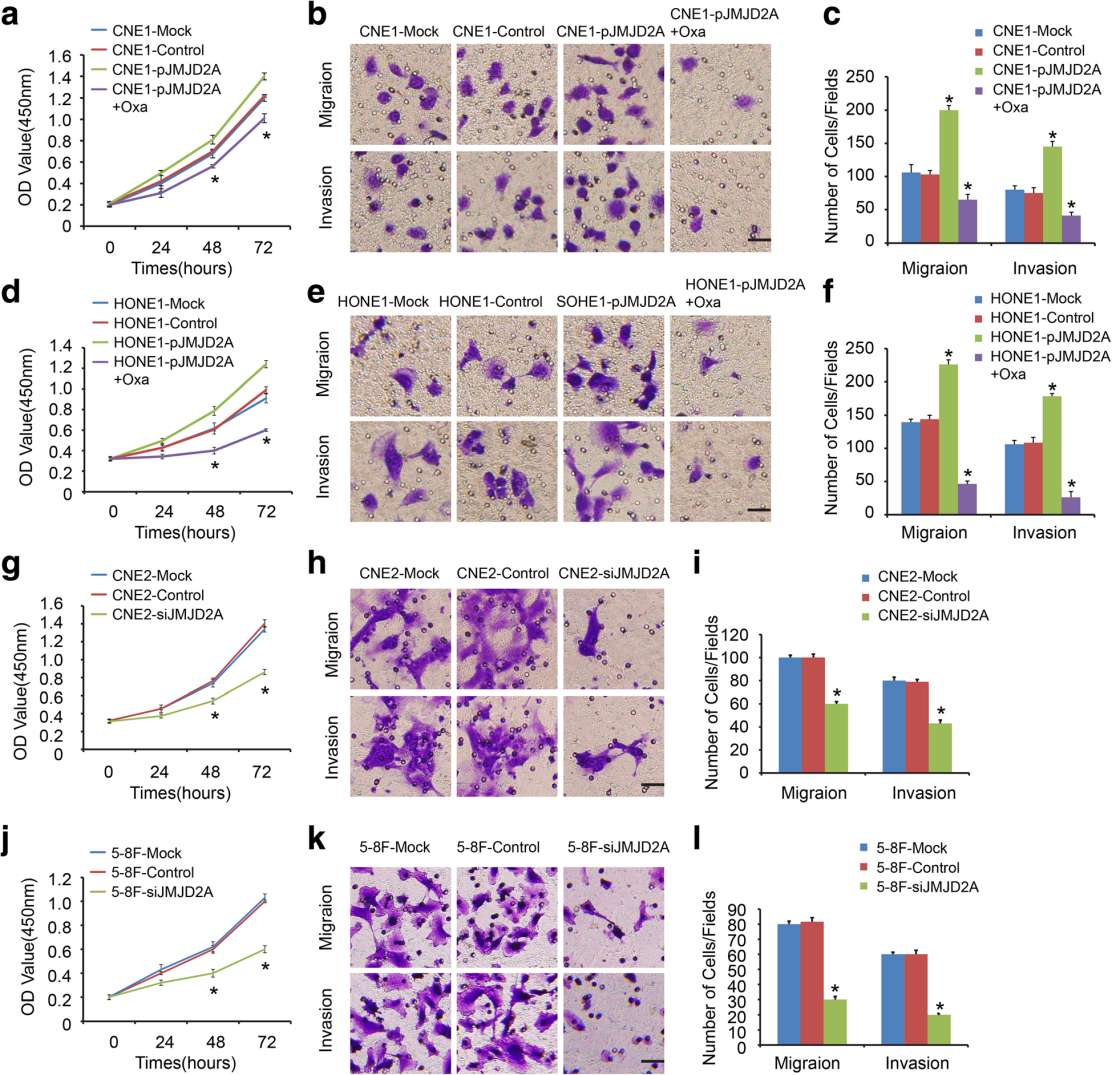
JMJD2A-LDHA signaling promoted NPC cell proliferation, migration and invasion. **a-f** JMJD2A overexpression significantly promoted cell growth, migration and invasion in CNE1 (A, B, C) and HONE1 (D, E, F) cell lines. Oxamate-treated CNE1 (A, B, C) and HONE1 (D, E, F) cells transfected with pJMJD2A grew slower and exhibited less migration than pJMJD2a cells. **g-l** Knockdown of JMJD2A in CNE2 (G, H, I) and 5-8F (J, K, L) cells exhibited reduced proliferation, migration and invasion. *P < 0.05

We overexpressed JMJD2A in CNE1 cells treated with or without oxamate to detect the effects of JMJD2A-LDHA signaling on the biological features of NPC. JMJD2A overexpression significantly promoted cell growth (Fig. 5a; *P* < 0.05), migration and invasion (Fig. 5b and c; *P* < 0.05). Oxamate-treated CNE1 cells transfected with pJMJD2A grew slower and exhibited less migration than pJMJD2a cells (Fig. 5a, b, and c; *P* < 0.05). These results were confirmed in the HONE1 cell line (Fig. 5d, e, and f; *P* < 0.05). Consistently, two siJMJD2A cell lines, CNE2 (Fig. 5g, h, and i; *P* < 0.05) and 5-8F (Fig. 5j, k, and l; *P* < 0.05), exhibited reduced proliferation, migration and invasion.

### Direct correlations between JMJD2A and LDHA expression with the pathologic features of NPC

**Fig. 6:**
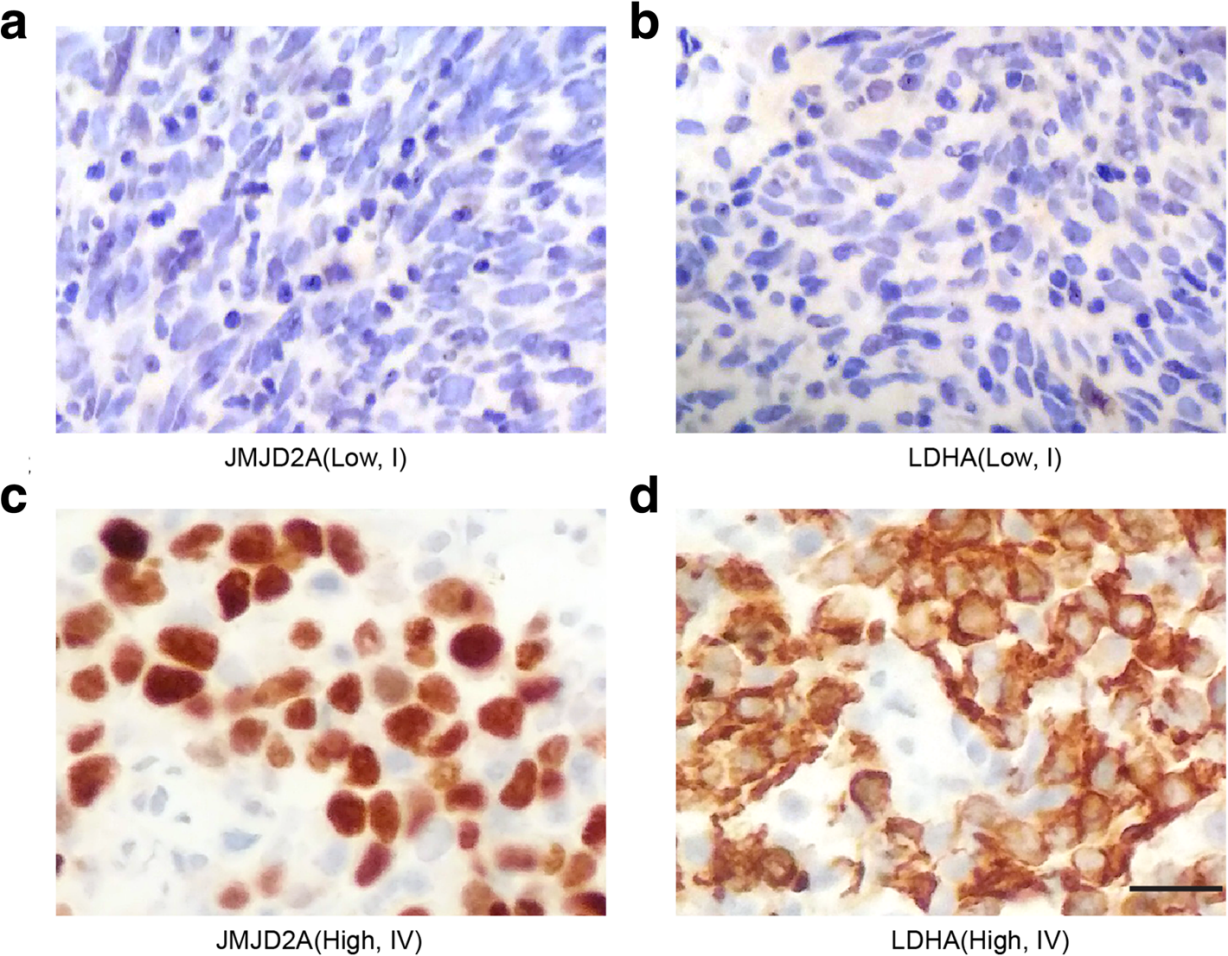
Immunohistochemical staining for the JMJD2A and LDHA proteins in NPC tissues at different clinical stages. Representative figures showed that JMJD2A and LDHA expression were positively correlated with advanced tumor stages. **a-b** Low JMJD2A and LDHA expression from one patient with a stage I tumor. **c-d** High JMJD2A and LDHA expression from another patient with a stage IV tumor

**Fig. 7:**
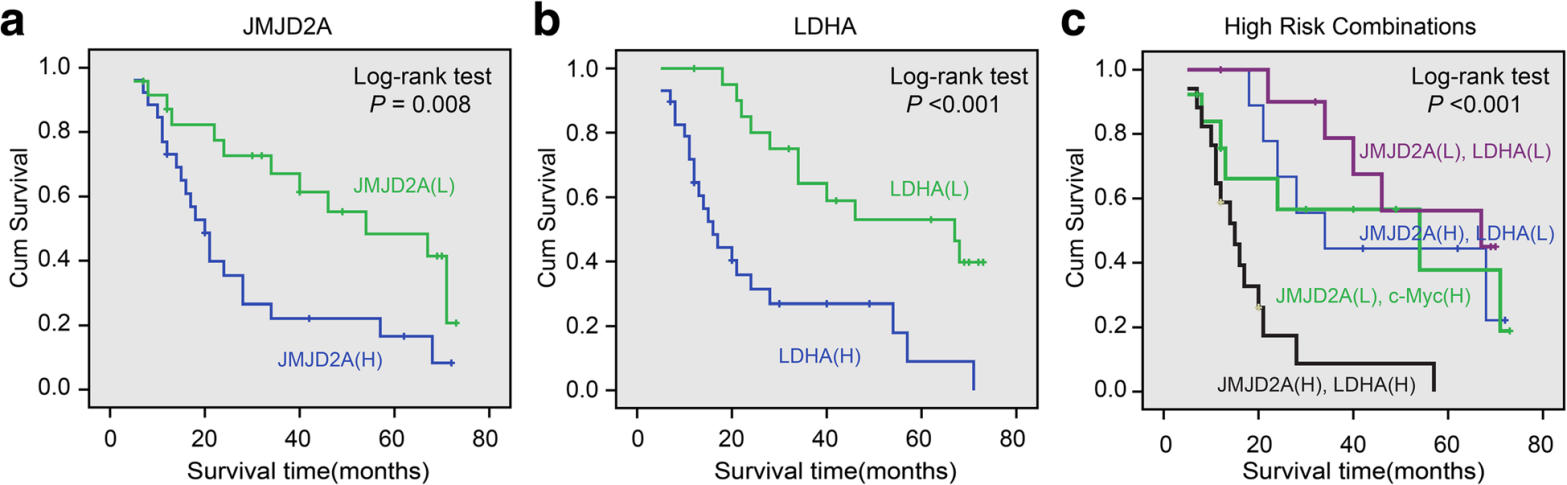
Kaplan-Meier analysis of the correlations between JMJD2A and LDHA expression with the prognosis of patients with NPC. **a** Patients with higher JMJD2A expression have a poor prognosis. **b** Patients with higher LDHA expression have a poor prognosis. **c** Patients with high expression of both JMJD2A and LDHA displayed the worst prognosis. “L” represents low, “H” represents high

We provided evidences that JMJD2A transcriptionally regulated LDHA gene expression and NPC glycolysis. We investigated JMJD2A and LDHA expression in NPC tumor specimens using IHC. The expression of both JMJD2A and LDHA was positively correlated with the T, M classification and clinical stage (Table 2; *P* < 0.05). As shown in representative figures, JMJD2A and LDHA expression were positively associated with advanced tumor stages (Fig. 6a, b, c, and d; *P* < 0.05). Additionally, the level of JMJD2A was positively correlated with LDHA expression in NPC tissues (Table 3, *r* = 0.642, *P* < 0.05). Further, we performed a Kaplan-Meier analysis and found that higher JMJD2A or LDHA expression predicted a worse prognosis (Fig. 7a and b; *P* < 0.05). Patients with higher expression of both JMJD2A and LDHA had the worst prognosis (Fig. 7c; *P* < 0.05). According to the Cox analysis, both JMJD2A and LDHA may be predictive markers for patients with NPC (Table 4; *P* < 0.05). Based on these data, JMJD2A-LDHA signaling regulates NPC development and progression.

**Table 2:** Associations between JMJD2A, LDHA protein expression and clinicopathological characteristics in NPC

Variable	Cases	JMJD2A expression		*P*-value	LDHA expression		*P*-value
		Low (*n* = 24)	High (*n* = 26)		Low (*n* = 21)	High (*n* = 29)	
Gender							
Male	22	10	12	0.749	9	13	0.890
Female	28	14	14		12	16	
Age(years)							
< 50	21	9	12	0.536	7	14	0.291
≥ 50	29	15	14		14	15	
Histological type							
DNKC	25	11	14	0.571	10	15	0.774
UDC	25	13	12		11	14	
T classification							
T1-T2	31	20	11	0.003*	18	13	0.003*
T3-T4	19	4	15		3	16	
N classification							
N0-N1	32	14	18	0.423	13	19	0.793
N2-N3	18	10	8		8	10	
M classification							
M0	37	21	16	0.037*	19	18	0.024*
M1	13	3	10		2	11	
Clinical stage							
I-II	24	17	7	0.002*	16	8	0.001*
III-IV	26	7	19		5	21	

*DNKC* differentiated non-keratinizing carcinoma, *UDC* undifferentiated carcinoma, *T* tumor size, *N* lymph node metastasis, *M* distant metastasis

*P < 0.05 indicates a significant association among the variables (2-tailed)

**Table 3:** Correlation analysis between JMJD2A and LDHA protein expression in NPC

Tissue sample	LDHA expression		r	*P*-value
	Low	High		
JMJD2A Low	18	6	0.642	<0.001*
JMJD2A High	3	23		

*P < 0.05 indicates that correlation is significant at the 0.05 level (2-tailed)

**Table 4:** Summary of univariate and multivariate Cox regression analysis of overall survival duration in all NPC patients (*n* = 50)

Clinicopathological parameters	Univariate analysis			Multivariate analysis		
	HR	95% CI	*P*-value	HR	95% CI	*P*-value
JMJD2A(High/Low)	2.539	1.233–5.228	0.011*	2.652	1.245–5.650	0.011*
LDHA(High/Low)	3.652	1.721–7.749	0.001*	3.433	1.560–7.556	0.002*
Gender (Female/Male)	2.324	0.954–5.662	0.063			
Age(years)(≥50/<50)	0.681	0.341–1.357	0.681			
Histological type(UDC/DNKC)	0.614	0.308–1.224	0.166			
T classification(T3 + T4/T1 + T2)	1.351	0.679–2.687	0.391			
N classification (N2 + N3/N0 + N1)	2.941	1.160–7.455	0.023*			
M classification (M1/M0)	2.349	1.107–4.984	0.026*			
Clinical stage (III + IV/I + II)	2.291	1.096–4.788	0.028*			

HR hazard ratio, 95% CI 95% confidence interval, *indicates *P* < 0.05

## Discussion

In our research, we have investigated the role of JMJD2A in NPC metabolism and JMJD2A-LDHA signaling in NPC tumorigenesis. We provided evidences supporting a critical role for JMJD2A in the glycolysis regulation via the transcriptional activation of LDHA gene expression. First, JMJD2A was upregulated in NPC tumor tissues and cell lines. Second, JMJD2A silencing decreased the LDHA expression at mRNA and protein level and increased LDH activity, glucose utilization, lactate production, and the ATP level, and JMJD2A overexpression had the opposite effects. Third, JMJD2A directly bound the LDHA promoter region and transcriptionally regulated LDHA gene expression. Fourth, activated JMJD2A-LDHA signaling pathway promoted NPC cell proliferation, migration and invasion. Additionally, both JMJD2A and LDHA expression were positively correlated with the TNM classification and clinical stage. Moreover, JMJD2A expression was positively correlated with LDHA expression in NPC tissues, and higher JMJD2A and LDHA expression predicted a worse prognosis. Thus, JMJD2A regulates glycolysis in NPC by modulating LDHA expression. This novel JMJD2A-LDHA signaling pathway could contribute to the Warburg effect in NPC cells and tumorigenesis and progression.

JMJD2A, as a histone demethylase, plays vital role in various cancer types. Its function as an oncogene or suppressor gene remains unclear. In bladder cancer, JMJD2A is expressed at significantly lower levels in cancer samples than in normal tissues [21]. Lower JMJD2A expression is correlated with a poorer prognosis [21]. However, JMJD2A is upregulated at malignant gastric cancer tissues in comparison with that of normal control. JMJD2A regulates gastric cancer cell growth and serves as an independent prognostic factor [18]. Furthermore, JMJD2A participates in carcinogenesis by regulating the G1/S transition in lung cancers and bladder cancers [19]. Additionally, JMJD2A is overexpressed in breast cancer [9, 10, 22], lung cancer [11, 19], prostate cancer [23], colorectal cancer [24], and head and neck squamous cell carcinoma [25]. In our study, we first observed JMJD2A overexpression in human NPC and showed that it was correlated with the TNM classification and clinical stage, promoting NPC progression.

We have revealed a critical role for JMJD2A in NPC progression, but the previous studies provide little evidence of revealing JMJD2A function in the cancer metabolism. The Warburg effect is considered a hallmark of cancer [12]. The Warburg effect means that tumor cells predominantly produce energy through glycolysis and followed by the lactic acid fermentation [26], rather than through a comparatively low level of glycolysis rate, followed by the oxidation of pyruvate in mitochondria, as occurs in most normal cells [27–29]. Upregulated enzymes and glucose transporters of glycolytic pathway as the results of oncogene activation are main reasons for the Warburg effects [30]. In our study, JMJD2A silencing did not alter the most glycolytic enzymes expression, with the exception of PFK-L, PGAM-1, LDHB and LDHA. Because LDHA exhibited the greatest decrease in expression and mainly converts pyruvate to lactate, we mainly explored the function of JMJD2A in glycolysis through activating LDHA expression in this study.

LDHA, which catalyzes the last step of anaerobic glycolysis, is a major subunit of LDH. Abnormal LDHA expression is universal in many human cancers, such as pancreatic cancer [31], hepatocellular carcinoma [32], and breast cancer [33], suggesting that the overexpression of this gene promotes cancer development and progression. LDHA is reported to be an adverse independent prognostic factor for NPC [34]. In our present study, the LDHA level was elevated, and correlated with the TNM and clinical stage in NPC tissue samples. Based on these results, the level of LDHA expression was associated with NPC development and progression. Moreover, LDHA was positively correlated with JMJD2A expression in tumor specimens. Next, we evaluated whether altering JMJD2A expression could exert effects on LDHA expression, LDH activity, glucose utilization, lactate production, and the intracellular ATP level. JMJD2A overexpression markedly increased LDHA expression, LDH activity, glucose utilization, and lactate production, and decreased the intracellular ATP level, whereas JMJD2A knockdown had the opposite effects. Thus, JMJD2A influences the Warburg effect by regulating LDHA expression. A major focus of JMJD2A studies was its role in transcriptional regulation, where it may either activate or inactivate genetic transcription. The latter function may involve the correlation with histone deacetylases or the nuclear receptor co-repressor complex [35, 36] or direct binding to a transcription factor, as observed for the p53 gene [37]. We further studied whether JMJD2A could regulate LDHA expression by modulating through transcriptional level. In the present study, JMJD2A directly bound to LDHA promoter region and activated LDHA expression transcriptionally. However, the detailed molecular mechanisms by which JMJD2A regulates LDHA expression require further exploration. Next, we analyzed the effects of JMJD2A-LDHA signaling alterations on NPC cell growth and invasion in vitro. Elevated JMJD2A-LDHA signaling promotes cell proliferation and invasion, whereas decreased JMJD2A-LDHA signaling had the opposite effects. Taken together, our results have implied that JMJD2A regulates the Warburg effect in NPC by transcriptionally regulating LDHA expression.

## Conclusions

In summary, our study is the first to share some critical insights into the role of JMJD2A in NPC glycolysis metabolism and identified a role of a novel JMJD2A-LDHA signaling in NPC tumorigenesis. We identified and demonstrated a novel JMJD2A-LDHA signaling pathway alteration, which could be promising molecular target for new therapeutic exploration to control NPC.

## Additional file

Additional file 1: Figure S1A. Three primers targeting the LDHA promoter region. **B.** ChIP assay using chromatin isolated from CNE2 cells, and primer b showed the largest difference **P* < 0.05. (TIFF 502 kb)

## Abbreviations

ChIP: Chromatin immunoprecipitation
JMJD2A: Jumonji C domain 2A
MTT: 3-(4,5-dimethylthiazol-2-yl)-2,5-diphenyl-tetrazolium bromide
NPC: Nasopharyngeal carcinoma
