# Supplementary figures and images for: JMJD2A promotes the Warburg effect and nasopharyngeal carcinoma progression by transactivating LDHA expression
Source: BMC Cancer. 2017 Jul 11;17:477. doi: 10.1186/s12885-017-3473-4 (PMC5504777; doi:10.1186/s12885-017-3473-4)

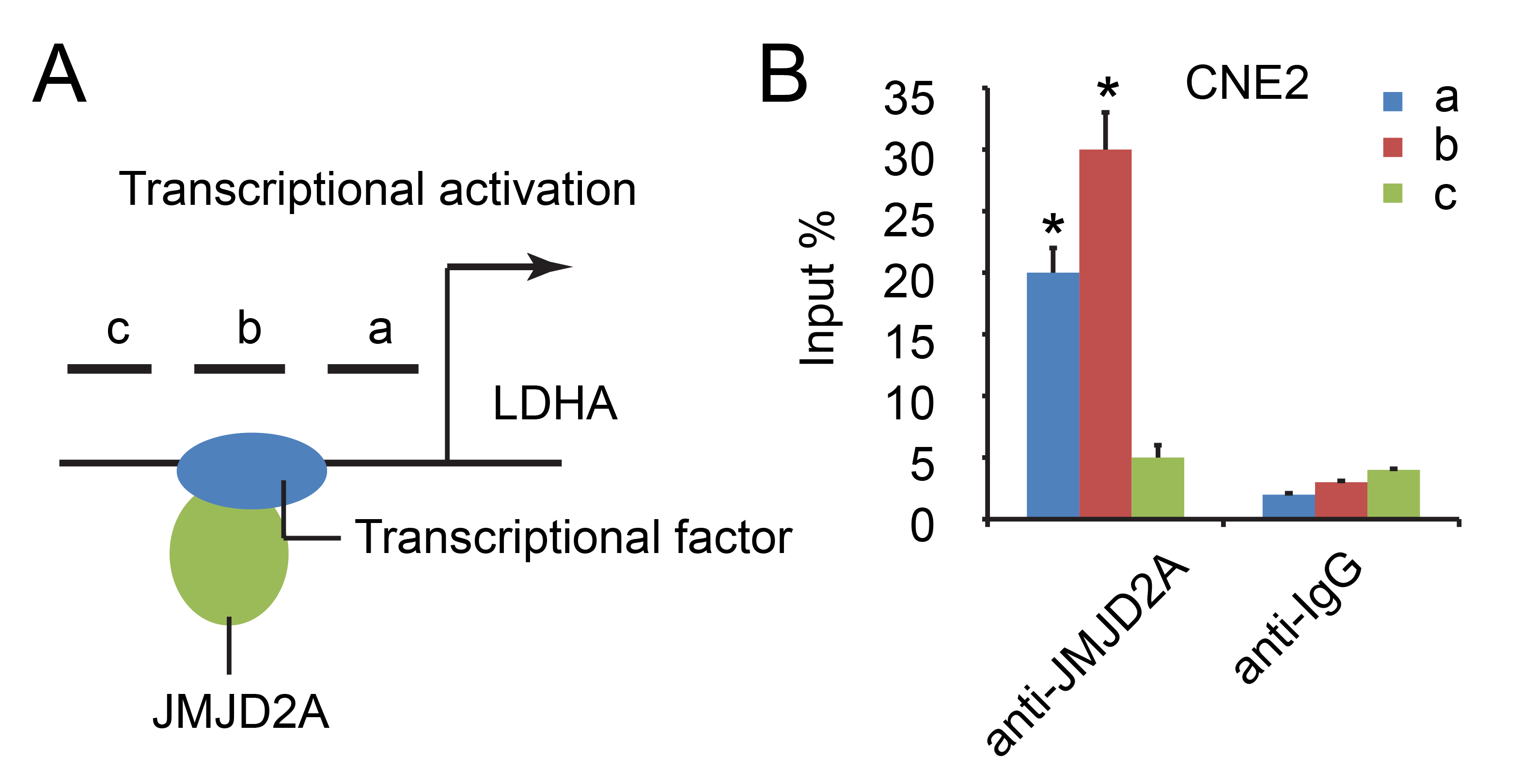

Supplement: Additional file 1: Figure S1A. — Three primers targeting the LDHA promoter region. B. ChIP assay using chromatin isolated from CNE2 cells, and primer b showed the largest difference *P < 0.05. (TIFF 502 kb) [file 12885_2017_3473_MOESM1_ESM.tif]
